# Comment on “Comparing Physician Assistant and Nurse Practitioner Practice in US Emergency Departments, 2010–2017”

**DOI:** 10.5811/westjem.2022.3.56163

**Published:** 2022-09-19

**Authors:** Sean Croughan, Deirdre Glynn

**Affiliations:** St. James Hospital, Department of Emergency Medicine, Dublin, Ireland

To the Editor:

We read with great interest the piece by Wu and colleagues, which explores the changing landscape of emergency medicine and increasing use of non-physician healthcare professionals in recent years.^1^ We applaud the tremendous efforts of the authors to provide much needed quantitative data on a topic that is likely to become increasingly important. The paper raised great interest locally; Ireland has a nascent nurse practitioner programme but is also tentatively exploring physician assistant education models. Data such as this is invaluable to help management make informed decisions regarding future workforce planning.

However, we feel there are some important methodological issues that need to be considered to fully evaluate the value of the authors’ data. In the data analysis subsection of the manuscript, no details are provided of the statistical methodology that is used to compare between-group differences. From this, we assume that the authors simply computed confidence intervals for each measurement and then compared these intervals. This technique is often overly conservative, substantially increasing the risk of a type II error.^2^ The error arises because instead of considering the confidence interval of the difference between means (the value we are actually interested in), we are comparing the confidence intervals of each mean (separate and distinct values). A more robust method would be to perform a hypothesis test to determine the between-group difference and report the confidence intervals of this inter-group difference. This approach also allows for easy assessment of the magnitude of the inter-group difference, if any, to determine whether the effect size is clinically meaningful or merely statistically significant.

We would note that for the comparison between “physician assistant (PA) with physician involvement” and “nurse practitioner (NP) with physician involvement”, along with certain other measurements, a *P*-value is provided. This suggests this technique may have been used; but there is no data in the manuscript to describe what testing methodology the *P*-value refers to.

Without this data, interpretation of the study is limited as we are unable to confidently exclude true inter-group differences. This becomes even more important when considering that clinically relevant differences in variables, such as frequency of diagnostic testing, imaging, procedures performed, medications prescribed and admission rates, may have been overlooked due to these methodological issues. However, it should be noted that applying previously described estimation techniques suggest no true inter-group difference.^3^

We would like to thank the authors for taking the time to produce a work on such an interesting and important topic to the future of emergency medicine. If the authors could expand upon the concerns highlighted above, we feel that this would greatly increase the usefulness of the data provided.
